# Transcriptome Profiles Reveal the Growth-Promoting Mechanisms of *Paenibacillus polymyxa* YC0136 on Tobacco (*Nicotiana tabacum* L.)

**DOI:** 10.3389/fmicb.2020.584174

**Published:** 2020-09-25

**Authors:** Hu Liu, Jun Wang, Huimin Sun, Xiaobin Han, Yulong Peng, Jing Liu, Kai Liu, Yanqin Ding, Chengqiang Wang, Binghai Du

**Affiliations:** ^1^College of Life Sciences, Shandong Engineering Research Center of Plant-Microbial Restoration for Saline-Alkali Land, Shandong Key Laboratory of Agricultural Microbiology, National Engineering Laboratory for Efficient Utilization of Soil and Fertilizer Resources, Shandong Agricultural University, Tai’an, China; ^2^Zunyi Tobacco Monopoly Administration of Guizhou, Zunyi, China

**Keywords:** *Paenibacillus polymyxa*, tobacco, PGPR, growth-promoting mechanisms, RNA-seq

## Abstract

*Paenibacillus polymyxa* is an important member of the plant growth-promoting rhizobacteria. *P. polymyxa* YC0136 inoculation had beneficial effect on growth promotion and biological control of tobacco (*Nicotiana tabacum* L.) under field conditions. This study aimed to reveal the growth-promoting mechanisms of strain YC0136. In growth-promotion assays, tobacco plant height was increased by 8.42% and 8.25% at 60 and 90 days, respectively, after inoculation with strain YC0136. Strain YC0136 also promoted the accumulation of tobacco biomass in varying degrees. Following inoculation with strain YC0136, 3,525 and 4,368 tobacco genes were up-regulated and down-regulated, respectively. Strain YC0136 induced the expression of plant hormone-related genes in tobacco, including auxin, cytokinin, and gibberellin, as well as transcription factors related to stress resistance such as WRKY and MYB. In addition, strain YC0136 induced the up-regulation of genes in the phenylpropanoid biosynthesis pathway by 1.51–4.59 times. Interaction with tobacco also induced gene expression changes in strain YC0136, with 286 and 223 genes up-regulated and down-regulated, respectively. Tobacco interaction induced up-regulation of the *ilvB* gene related to auxin biosynthesis in strain YC0136 by 1.72 times and induced expression of some nutrient transport genes. This study contributes to our understanding of the growth-promoting mechanisms of strain YC0136 on tobacco and provides a theoretical basis for the application of *P. polymyxa* YC0136 as a biological fertilizer.

## Introduction

The numerous microorganisms in the rhizosphere of plants can be divided into positive, negative, and neutral microorganisms. Plant growth-promoting rhizobacteria (PGPR) are a group of rhizobacteria colonizing the plant rhizosphere that can promote the growth of many plants (Salvo et al., 2018). In modern agriculture, the use of pesticides and chemical fertilizers is becoming more limited, and PGPR are attracting more and more attention. PGPR can not only be used as plant growth regulators but also as biological control agents (Recep et al., 2009; Bal et al., 2013a; Zhang and Kong, 2014; Djaya et al., 2019). PGPR’s associated with diverse plant hosts including tobacco variety K326 (Zhang and Kong, 2014), sudan grass var *Sudanensis* (Basak and Biswas, 2009), rice (cv. Naveen) (Bal et al., 2013a), potato cultivars Agria and Granola (Recep et al., 2009), cucumber (Han and Lee, 2006), common bean (Mohamed et al., 2019), *Helianthus annuus* (Khan et al., 2018), wheat (Mohite, 2013), and *Arabidopsis thaliana* (Jiang et al., 2012). PGPR promote growth through direct and indirect mechanisms (Santoro et al., 2015). Direct mechanisms include secretion of auxin (Lata et al., 2006) or cytokinin (Timmusk et al., 1999), phosphate solubilization (Son et al., 2006), dissolving potassium (Basak and Biswas, 2009), nitrogen fixation (Anand et al., 2013), siderophore production (Sarode et al., 2007), and 1-aminocyclopropane-1-carboxylate (ACC) deaminase production (Bal et al., 2013b). PGPR can not only promote plant growth directly, but can also be used as biocontrol agents to control plant pathogens, such as *Aspergillus niger* (Yuttavanichakul et al., 2012), *Candida albicans* (Ajilogba et al., 2016), *Fusarium*. sp. (Cao et al., 2018), *Armillaria* sp. (Vasconcellos and Cardoso, 2009), and *Cercospora arachidicola* (Sayyed and Patel, 2011). Inhibiting the growth of pathogens by biosynthesizing secondary metabolites is a major indirect growth-promoting mechanism of PGPR. The secondary metabolites are various, such as phenazines (Burkhead et al., 1994), iturin A (Ahimou et al., 2000), bacillomycin (Volpon et al., 1999), polymyxin (Kagan et al., 1951; Cruz et al., 2007; Niu et al., 2013), and fusaricidin (Vater et al., 2016). Inducing resistance in plants is another important indirect growth-promoting mechanism of PGPR (Tahir et al., 2017).

*Paenibacillus polymyxa*, formerly known as *Bacillus polymyxa*, was distinguished from other members of the genus *Bacillus* using a highly specific gene probe based on 16S rRNA (Ash et al., 1993). *P. polymyxa* is an important member of the PGPR that can promote the growth of many hosts through direct or indirect mechanisms (Michael et al., 1997; Ali, 2010; Muthukumar and Udaiyan, 2010; Lee et al., 2012; Anand et al., 2013; Kim et al., 2016; Abdallah et al., 2019). Research by Puri et al. (2016) suggested that *P. polymyxa* P2b-2R could increase the biomass and seedling height of canola through nitrogen fixation. *P. polymyxa* BRF-1 increased soybean dry weight and mineral absorption and has biocontrol ability against *Phialophora gregata* (Zhou et al., 2010). *P. polymyxa* ICA B01, a strain with phosphate solubilizing characteristic *in vitro*, promoted the growth of *Zea mays* under abiotic stress conditions (Mohd Din et al., 2020). Phi et al. (2010) reported that *P. polymyxa* KNUC265 improves the yield of pepper and antagonizes the pathogen *Erwinia carotovora* subsp. *carotovora* in tobacco. *P. polymyxa* ATCC 842^T^ promoted the growth of tomato by producing indole-3-acetic acid (IAA) and inducing systemic resistance (Mei et al., 2014). *P. polymyxa* is a valuable species for agricultural application and has important research value.

Beneficial plant-microbe interactions that promote plant health and development have been the subject of considerable study. *Azospirillum brasilense* REC3, a PGPR strain, promoted the growth of strawberry plants (Elías et al., 2018), and the up-regulation of genes associated with ethylene (ET) signaling and IAA biosynthesis was detected by fluorescence quantitative PCR. Lee et al. (2012) demonstrated that *P. polymyxa* E681 elicited the induced systemic resistance (ISR) and plant growth promotion of *Arabidopsis* by producing C13 volatiles. Molecular aspects of the *Gluconacetobacter diazotrophicus-*sugarcane interaction have been investigated by quantitative mass spectrometry-based proteomics (Lery et al., 2010). Inoculation with PGPR strains could affect the transcriptomic profiles of tomato (Ibort et al., 2018). In Ibort’s research, *Enterobacter* sp. strain C7 alleviated the stress level of water through reducing the expression of ethylene biosynthesis gene in wild-type tomato. *B. megaterium* inoculation induced the expression of ethylene biosynthesis, signaling, and response genes in *never ripe* plants. In addition, *B. megaterium* could enhance the antioxidant capacity of wild-type tomato through affecting the expression of flavonoid biosynthesis genes. Plants also exert influence on microorganisms. Transcriptome profiling of *P. aeruginosa* PA01 response to sugar beet root exudates revealed that root exudates affected the expression of genes related to metabolism, chemotaxis, and other processes (Mark et al., 2005).

*P. polymyxa* YC0136 is a PGPR strain isolated from tobacco rhizosphere soil in Zunyi, Guizhou Province, China. It promotes the growth of tobacco and inhibits the growth of some tobacco pathogens, such as *Ralstonia solanacearum*, *Phytophthora parasitica* var. *nicotianae* (data not shown). In this study, we carried out RNA-seq of tobacco roots co-cultured with YC0136 cells under sterile conditions to analyze the promotion mechanism of strain YC0136 on improving the agronomic traits of tobacco and enhancing the resistance of tobacco. The effect of tobacco on gene expressing of strain YC0136 was also discussed.

## Materials and Methods

### Strains, Plant Material, and Growth Conditions

*P. polymyxa* YC0136 was previously isolated using Luria-Bertani (LB) agar medium from the rhizosphere soil of tobacco in Zunyi and stored in the PGPR laboratory, Shandong, China. Strain YC0136 was grown on Luria-Bertani (LB) agar medium (Ma et al., 2009) and cultured overnight at 37°C. A pure culture of strain YC0136 was grown in 5 mL of LB liquid medium for 12 hours (h) at 37°C and 180 rpm. Then 5 mL of the culture was transferred into 50 mL of fresh LB liquid medium and shaken for another 12 h at 37°C. The cell suspensions (diluted to ∼10^8^ cells per milliliter) were used for growth promotion assays in pots. In addition, YC0136 cells cultured under the same conditions were harvested, washed with sterile water, and diluted to optical density OD_600_ = 0.9. These cells were used for transcript profiling of strain YC0136 and tobacco under sterile conditions.

*Ralstonia solanacearum* and *Fusarium moniliforme* were used for antagonistic tests. A pure culture of *R. solanacearum* was incubated in 5 mL of LB liquid medium for 10 h at 37°C with shaking (180 rpm). *F. moniliforme* was activated on Potato Dextrose Agar (PDA) medium (Cardwell et al., 2000) and cultured for 4 days, at 28°C.

Tobacco cultivar *Nicotiana tabacum* L. ‘K326’ was selected as plant material. It is a type of flue-cured tobacco widely planted in China.

### Determination of Plant Growth-Promoting Traits

Preparation of cultures for detection of auxin secretion followed the method of Shao et al. (2015). Strain YC0136 was inoculated into Landy liquid medium with L-tryptophan and cultured at 25°C and 90 rpm with shaking for 4 days. Salkowski colorimetric solution was used to detect IAA secretion ability, and the absorbance of the mixture at 530 nm was measured (Thakuria et al., 2004), with 50 mg/L IAA used as a positive control. The negative control was bacteria-free Landy medium with L-tryptophan. IAA production was determined by the standard curve method (Yu et al., 2016).

Organophosphorus culture medium (Qingdao Hope Bio-technology Co., Ltd.) was used to test the phosphate solubilization ability of strain YC0136. Strain YC0136 was inoculated on organophosphorus medium and cultured at 30°C for 7 days until a transparent circle formed around the colonies. The ratio of transparent circle diameter (*D*)/colony diameter (*d*) indicated the capacity for phosphate solubilization (Kanse et al., 2015).

The ability of strain YC0136 to antagonize bacteria and fungi was also tested. The plant pathogens (*R. solanacearum* and *F. moniliforme*) were selected as indicators in the antagonistic test of this study. One hundred microliters of *R. solanacearum* culture were coated on LB agar medium, then strain YC0136 was inoculated on the same plate. The strains were co-cultured at 37°C for 24 h. Agar containing *F. moniliforme* was placed at the center of PDA agar medium plates and cultured for 2 days at 28°C. Strain YC0136 was then inoculated on the plate around the *F. moniliforme* (Príncipe et al., 2007). They were co-cultured at 28°C for another 2 days. The appearance of inhibition zones on the plates indicated that strain YC0136 had antagonistic abilities.

### Growth-Promotion Assay in Pots

To evaluate the plant growth-promoting effects exerted by strain YC0136 on tobacco, plant height, leaf number, and biomass of tobacco were determined. The assay was carried out in the greenhouse of Shandong Agricultural University (36.200796°N, 117.125149°E). For pot experiments, tobacco seeds were sown in nutrition soil (total nutrient content ≥3%, organic matter content ≥30%) and cultured at room temperature. Tobacco seedlings with 5–6 leaves were planted in pots (diameter 22 cm, depth 15 cm) with 3 kg of soil per pot. Two hundred milliliters of water containing 5 mL of strain YC0136 cell suspension was poured into the rhizosphere of tobacco plants. There were 10 biological replicates in each group. At the same time, the control group was treated with 200 mL of water with 5 mL of LB medium. Plant height and leaf number were investigated every 30 days. For the investigation of plant height and leaf numbers, 10 plants were investigated at 30 days and 60 days after inoculation, and five plants were investigated at 90 days. At 60 days after strain inoculation, three plants in each group were collected for biomass analyses. These plants were dried at 105°C for 30 min, then dried at 80°C to constant weight.

### Interaction Between Strain YC0136 and Tobacco Under Sterile Conditions

For transcriptome tests, seeds of ‘K326’ were surface-sterilized and sown on 1/2 Murashige-Skoog (MS) medium for germination (Timmusk et al., 2005). Tobacco was grown in a chamber at 25°C with 10 h light period (13200 lux) and 14 h dark period. Seven-day-old seedlings were used for subsequent growth experiments. Tobacco seedlings were transplanted into tissue culture bottles with 90 mL of liquid MS medium. The tissue culture bottles contained a net to support the seedlings and a tube to support the net. Tobacco was grown in a chamber at 25°C with 10 h light period (13200 lux) and 14 h dark period for 30 days. One milliliter of YC0136 cells was then inoculated into each tissue culture bottle for co-culturing with the tobacco. The tobacco control group was inoculated with 1 mL of sterile water. Meanwhile, 1 mL of YC0136 cell suspension was inoculated in a bottle with MS medium but without tobacco as the control group for strain YC0136. Each treatment contained three biological replicates. Strain YC0136 and tobacco ‘K326’ were co-cultured for 20 h. Co-cultured YC0136 cells were then centrifuged at 8000 rpm for 2 min and rinsed three times with sterile PBS buffer to completely remove impurities. YC0136 cells in the YC0136-tobacco co-cultured group were labeled YB. YC0136 cells in the control group were labeled YB-CK. Tobacco roots were harvested and washed with sterile water three times. Tobacco roots from the co-cultured group were labeled YP, and tobacco roots from the control group were labeled YP-CK. Harvested bacteria and tobacco roots were placed in liquid nitrogen for 3 h, then stored in a freezer at −80°C for future tests.

### Total RNA Extraction and Transcriptomic Analysis

Total RNAs of strain YC0136 and tobacco roots were extracted and purified using TRIzol Reagent (Invitrogen). The quality and quantity of RNAs were investigated using an Agilent 2100 (Agilent Technologies, Palo Alto, CA, United States) and NanoDrop (Thermo Fisher Scientific Inc.), and RNAs with RNA Integrity Number (RIN) > 7 were selected to construct the library. Next-generation sequencing library preparations were constructed according to the manufacturer’s protocol (NEBNext^®^ Ultra^TM^ RNA Library Prep Kit for Illumina^®^). Libraries with different indices were multiplexed and loaded on an Illumina HiSeq instrument according to the manufacturer’s instructions (Illumina, San Diego, CA, United States). Sequencing was carried out using a 2 × 150 bp paired-end configuration. Image analysis and base calling were performed using the HiSeq Control Software (HCS) + OLB + GAPipeline-1.6 (Illumina) on the HiSeq instrument.

Raw data were stored in fastq file format. Low-quality data and adaptor sequences were filtered out to ensure the accuracy of data. The complete genome of strain YC0136 (GenBank accession number: CP017967.3) was selected as the reference genome. Although a draft genome of tobacco ‘K326’ is available, many genes have no annotation information. The genome of *Nicotiana tabacum* L. cultivar ‘TN90’ was therefore selected as the reference genome^1^. Clean data were aligned to the reference genome using the software Hisat2 (v2.0.1) (Kim et al., 2018). HTSeq (v0.6.1) was used to estimate gene and isoform expression levels from the paired-end clean data (Anders et al., 2015). Differentially expressed genes (DEGs) were analyzed using DESeq2 (V1.6.3) in the Bioconductor software package (Love et al., 2014). Kyoto Encyclopedia of Genes and Genomes (KEGG^2^) pathway enrichment analysis of DEGs was performed using KOBAS (Song et al., 2015).

### RT-qPCR Assay for DEGs in RNA-Seq

One microgram of purified total RNA was used as template for first-strand cDNA synthesis using an *Evo M-MLV* RT Kit with gDNA Clean for qPCR (Accurate Biotechnology (Hunan) Co., Ltd.). Several genes identified through RNA-seq were selected for amplification using SYBR green qPCR. Primers were designed using Beacon Designer 7 and were listed in Supplementary Table S1. The tobacco *actin* gene was selected as a reference gene. In strain YC0136, the *GAPDH* gene (encoding glyceraldehyde-3-phosphate dehydrogenase) was used as the reference gene. Relative expression levels were calculated using the 2^–(delta – delta Ct)^ method (Livak and Schmittgen, 2001). Three biological replicates were performed.

### Statistical Analysis

The statistical analyses of agronomic characters used *t*-tests in GraphPad Prism 7 (GraphPad Software, San Diego, CA, United States), and *P* < 0.05 means that there was a significant difference. Due to the presence of plant sampling in the tests, there were no fewer than three replicates per statistical analysis. Columns were drawn using GraphPad Prism 7.

## Results

### General Characteristics of Strain YC0136

**FIGURE 1:**
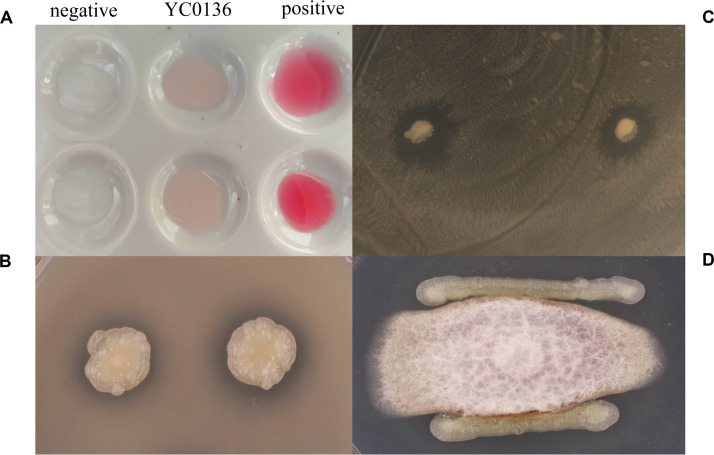
Growth-promoting characteristics of strain YC0136. **(A)** Detection of IAA secretion. A single clone of strain YC0136 was inoculated in Landy liquid medium and cultured for 4 days at 25°C, 90 rpm. Salkowski solution was then used to detect IAA secretion. A standard solution with 50 mg/L IAA was used as a positive control. Landy medium without inoculation was used as a negative control. **(B)** Qualitative analysis of phosphate solubilization. A single clone of strain YC0136 was cultivated on LB medium at 37°C. The pure bacterial culture was inoculated onto organophosphorus culture medium for qualitative analysis of phosphate solubilization. **(C)** Strain YC0136 inhibits the growth of *R. solanacearum. R. solanacearum* was coated on LB ager medium, and then strain YC0136 was inoculated onto the coated plate and cultured for 24 h at 37°C. **(D)** Strain YC0136 inhibits the growth of *F. moniliforme.* Hyphae of *F. moniliforme* were placed in the center of PDA medium and cultured for 2 days at 28°C. Strain YC0136 was then streaked around the fungi and cultured at 28°C for another 2 days.

We cultured strain YC0136 in Landy medium for 4 days before mixing with Salkowski colorimetric solution and incubating in the dark for 30 min. Figure 1A shows that the culture of strain YC0136 turned red. These indicated that strain YC0136 could secrete IAA. At an OD_600_ of 1.0, the IAA yield of strain YC0136 was 24 μg/mL. For analysis of phosphate solubilization, transparent circles (as shown in Figure 1B) generated around colonies of strain YC0136 indicated that strain YC0136 could dissolve organic phosphorus. The ratio *D/d* was 1.32 ± 0.04.

The results of the antagonistic assay are shown in Figures 1C,D. Strain YC0136 formed antagonistic circles on plates coated with *R. solanacearum*. This indicated that strain YC0136 can secrete antibacterial substances. There were obvious inhibitory bands on the edge of the *F. moniliforme* culture. This indicated that strain YC0136 can also inhibit the growth of fungi by secreting antifungal substances.

### Strain YC0136 Improves the Growth of Tobacco

**FIGURE 2:**
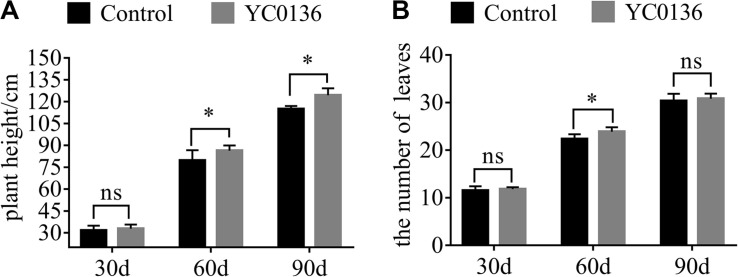
Effects of strain YC0136 on agronomic characteristics of tobacco at different growth stages. Two hundred milliliters of water with 5 mL of YC0136 cell suspension was poured into the rhizosphere of tobacco. In the control group, the cell suspension was replaced with 5 mL of LB medium. Plant height and leaf number were recorded every 30 days. **(A)** Statistical results for tobacco plant height. Plant height is the distance from the surface stem base to the first capsule base. **(B)** Statistical results for tobacco leaf number. Number of leaves is the number of leaves from the stem bottom to the top of the first flower branch. “^∗^” indicates a significant difference at the *p* < 0.05 level in *t*-tests. “ns” means that there is no significant difference at the *p* < 0.05 level. A total of two treatments were set up in the pot experiments with 10 replicates for each treatment at the beginning of the experiment. At 90 days after inoculation, there were five replicates. Error bars indicate the SD from the mean.

Agronomic traits of tobacco at different growth stages were promoted after inoculation with strain YC0136 (Figure 2). Strain YC0136 increased the plant height of tobacco at different growth stages (Figure 2A). At 30 days after inoculation, the plant height of tobacco in the group treated with strain YC0136 was slightly higher (3.79%) than that in the control group. There was a significant difference (*P* < 0.05) in the plant height of tobacco between the control group and the group treated with strain YC0136 at 60 days after inoculation. Compared with the control group, plant height in the group treated with strain YC0136 was increased by 8.42%. Moreover, the effects of strain YC0136 on tobacco plant height were also significant (*P* < 0.05) at 90 days after inoculation, with treated plants being 8.25% taller than controls. Strain YC0136 also increased the number of leaves on tobacco plants at different harvest intervals (Figure 2B). Plants treated with strain YC0136 had 6.97% more leaves than controls after 60 days of inoculation, and there was a significant difference at the *P* < 0.05 level.

**FIGURE 3:**
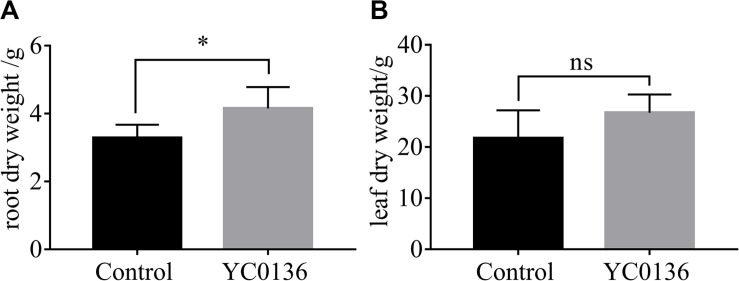
Effects of YC0136 on tobacco dry biomass at 60 days after inoculation. Plants were collected at 60 days after inoculation, dried at 105°C for 30 min, and then dried at 80°C to maintain constant weight. The data were analyzed by *t*-tests. The error bars were standard deviation of the group. **(A)** Statistical results for root dry weight at 60 days after inoculation. **(B)** Statistical results for leaf dry weight at 60 days after inoculation. Three replicates were used for statistical analysis. Error bars indicate the SD from the mean. “*” Indicates a significant difference at the *p* < 0.05 level in *t*-tests. “ns” Means that there is no significant difference at the *p* < 0.05 level.

At 60 days after inoculation, we sampled the tobacco plants and analyzed the dry biomass of the plants (Figure 3). Strain YC0136 had different effects on the biomass of different tobacco tissues. After 60 days of inoculation, root dry weight of tobacco plants in the group treated with strain YC0136 was increased by 26.5% compared to that in the control group, and the difference was significant at the *P* < 0.05 level (Figure 3A). Leaf dry weight was increased by 23% in the group treated with strain YC0136 compared with that in the control group, but this difference was not significant (Figure 3B).

### Transcriptome Profiling of the YC0136-Tobacco Interaction

**FIGURE 4:**
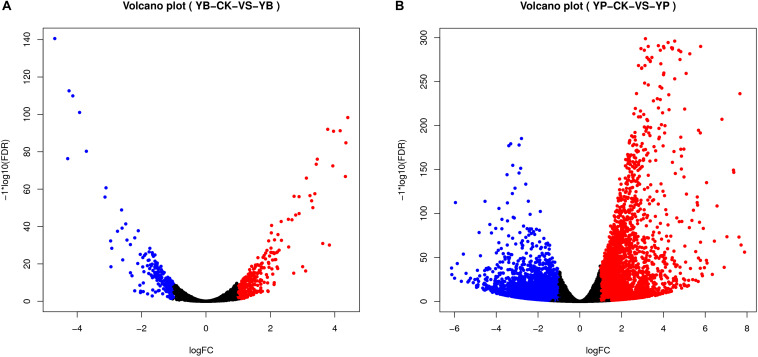
Visualization of differentially expressed genes (DEGs) in the interaction transcriptome (volcano map). DEGs were analyzed using DESeq2 (V1.6.3) in the Bioconductor software package and mapped to volcanoes. Red dots indicate gene up-regulation. Blue dots indicate gene down-regulation. The abscissa represents the fold change in gene expression in different samples. The ordinate represents the statistical significance of the difference in gene expression. **(A)** Visualization map of DEGs in strain YC0136. YC0136 cells in the YC0136-tobacco co-cultured group were labeled YB. YC0136 cells in the control group were labeled YB-CK. **(B)** Visualization map of DEGs in tobacco. Tobacco roots from the co-cultured group were labeled YP, and tobacco roots from the control group were labeled YP-CK.

We analyzed the transcriptomes of strain YC0136 and tobacco ‘K326’ during their interaction. A total of 286 genes were up-regulated and 223 genes were down-regulated in strain YC0136 under interaction conditions compared with control conditions (Figure 4A). Among these DEGs, 109 genes were significantly up-regulated (logFC > 1.5) and 78 genes were significantly down-regulated (| logFC| > 1.5) in strain YC0136. After interacting with strain YC0136, 3,525 genes in tobacco roots were up-regulated while 4,368 genes were down-regulated compared with those in uninoculated roots. The number of DEGs showing significant up-regulation (logFC > 1.5) was 2,041, and the number showing significant down-regulation (| logFC| > 1.5) was 2,423 (Figure 4B).

**FIGURE 5:**
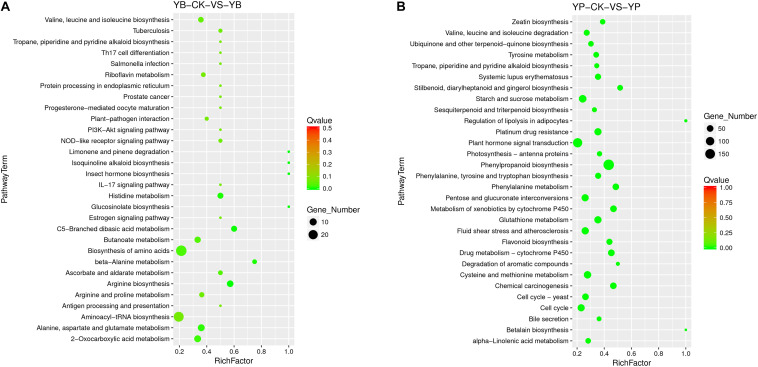
KEGG enrichment analysis of differentially expressed genes (DEGs) in the interaction transcriptome. KEGG enrichment of different genes was performed using KOBAS software. The degree of KEGG enrichment is measured by the Rich factor, the *Q*-value, and the number of genes enriched in this pathway. Rich factor refers to the ratio of the number of DEGs in the pathway to the total number of all annotated genes in the pathway. The larger the Rich factor, the greater the degree of enrichment. *Q*-value is the *p*-value after correction for multiple hypothesis testing. The value range of *Q*-value is [0,1]; the closer to zero, the more significant the enrichment. **(A)** KEGG enrichment of DEGs in strain YC0136. YC0136 cells in the YC0136-tobacco co-cultured group were labeled YB. YC0136 cells in the control group were labeled YB-CK **(B)** KEGG enrichment of DEGs in tobacco. Tobacco roots from the co-cultured group were labeled YP, and tobacco roots from the control group were labeled YP-CK.

We performed enrichment analysis of the DEGs in strain YC0136 using the KEGG database (Figure 5A). A total of 306 genes were enriched in 73 categories of four major categories in the KEGG database. Among the DEGs of strain YC0136, 38 genes were enriched in the ABC transporters pathway. There were 28 genes enriched in the biosynthesis of amino acids pathway. In tobacco, 1,797 DEGs were enriched in 52 pathways in four major KEGG categories (as shown in Figure 5B). Through analysis, we found that 183 genes were enriched in the phenylpropanoid biosynthesis pathway. Up to 130 genes were enriched in the signal transduction pathway of plant hormones. The number of genes related to the starch and sucrose metabolism pathway was 75. About 39 genes were enriched in the flavonoid biosynthesis pathway. Thirty-one genes were enriched in the terpenoid backbone biosynthesis pathway. Furthermore, 26 genes were enriched in zeatin biosynthesis.

#### Verification of Partial DEGs Using RT-qPCR

Reverse-transcription quantitative PCR is an important method for verifying gene expression in RNA-seq. To verify the accuracy of RNA-seq data, we selected some DEGs in tobacco and strain YC0136 for RT-qPCR.

**FIGURE 6:**
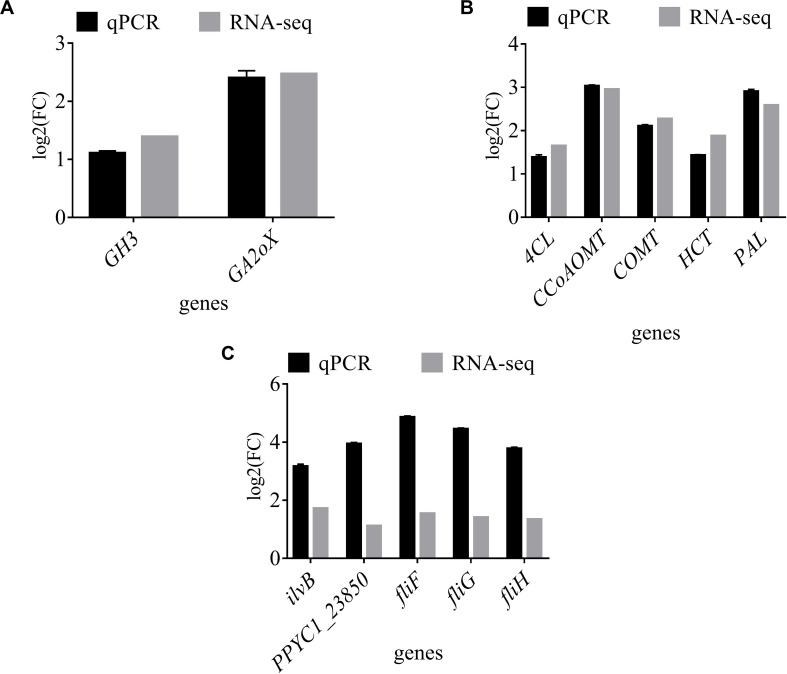
RT-qPCR assay of genes in tobacco and YC0136. Fluorescence RT-qPCR to verify the accuracy of the transcriptome data. One microgram of purified total RNA was used as template for first-strand cDNA synthesis. Several genes from RNA-seq were selected for amplification by SYBR green RT-qPCR. The *GAPDH* gene of strain YC0136 and the *actin* gene of tobacco were selected as references. Relative expression levels were calculated using the delta-delta Ct method. **(A)** RT-qPCR results for genes related to plant hormone transduction in tobacco. **(B)** RT-qPCR results for genes related to phenylpropane compound metabolism in tobacco. **(C)** RT-qPCR results for genes in strain YC0136. Three biological replicates were carried out, and three technical replicates were performed. Error bars indicate the SD from the mean.

RNA-seq of tobacco co-cultured with strain YC0136 revealed that genes (*GH3* and *GA2ox*) related to plant hormone transduction were up-regulated by 1.51–4.25 times and 1.57–4.98 times, respectively, compared with those in tobacco roots not cultured with strain YC0136. RT-qPCR showed that the expression of *GH3* (*gene56868*) and *GA2ox* (*gene14834*) was up-regulated by 1.11 and 2.40 times, respectively (Figure 6A). The gene expression trends revealed by RT-qPCR were consistent with those determined by RNA-seq. In RNA-seq, five genes (*4CL*, *CCoAOMT*, *COMT*, *HCT*, and *PAL*) related to metabolism of phenylpropane compounds were up-regulated by 1.63–1.75 times, 1.63–2.61 times, 2.26–4.59 times, 1.5–1.96 times, and 1.51–3.26 times, respectively, compared with those in tobacco roots not cultured with strain YC0136. We used these five genes (*gene65338*, *gene48930*, *gene51252*, *gene59104*, and *gene55404*) encoding *4CL*, *CCoAOMT*, *COMT*, *HCT*, and *PAL*, respectively, for RT-qPCR, and their expression was up-regulated by 1.39, 3.03, 2.11, 1.43, and 2.91 times, respectively (Figure 6B).

RT-qPCR results for strain YC0136 showed similar up-regulation of gene expression following interaction with tobacco as revealed by RNA-seq. Expression of *ilvB*, *PPYC1_23850*, *fliF*, *fliG*, and *fliH* was up-regulated by 3.17, 3.94, 4.85, 4.45, and 3.78 times, respectively, compared with that in strain YC0136 without tobacco roots (Figure 6C). The consistent trend in expression of these genes determined by RT-qPCR and RNA-seq indicated that the RNA-seq results were reliable.

#### Strain YC0136 Induces Plant Hormone Transduction in Tobacco

Early auxin response genes can be divided into three categories: *Aux/IAAs*, *GH3*, and *SAUR*s (small auxin up RNAs) (Liu et al., 2005). After inoculating with strain YC0136, *IAA*, *GH3*, and *SAUR* genes in tobacco were up-regulated by varying degrees (Table 1). Five genes (*gene28034*, *gene54165*, *gene2334*, *gene37942*, and *gene49814*) encoding SAUR family proteins were up-regulated by 1.73–2.59 times compared with those in tobacco roots not cultured with strain YC0136. Following stimulation by strain YC0136, expression of the *GH3* genes in tobacco increased by 1.51–4.25 times compared with that in tobacco not cultured with strain YC0136. The expression of auxin-responsive protein coding genes in tobacco roots was also up-regulated by 1.59–2.22 times.

**TABLE 1:** Effects of strain YC0136 on the genes related to auxin and gibberellin in tobacco.

Plant hormone	Gene ID	Log_2_(FC)	Gene	Product
IAA	*gene28034*	2.59	*SAUR*	SAUR family protein
	*gene54165*	2.20	*SAUR*	SAUR family protein
	*gene2334*	1.89	*SAUR*	SAUR family protein
	*gene37942*	1.84	*SAUR*	SAUR family protein
	*gene49814*	1.73	*SAUR*	SAUR family protein
	*gene4468*	4.25	*GH3*	Auxin-responsive GH3 gene family
	*gene65249*	1.69	*GH3*	Auxin-responsive GH3 gene family
	*gene11692*	1.63	*GH3*	Auxin-responsive GH3 gene family
	*gene15243*	1.51	*GH3*	Auxin-responsive GH3 gene family
	*gene7537*	2.22	*IAA*	Auxin-responsive protein IAA
	*gene32907*	1.90	*IAA*	Auxin-responsive protein IAA
	*gene10414*	1.74	*IAA*	Auxin-responsive protein IAA
	*gene1627*	1.59	*IAA*	Auxin-responsive protein IAA
Gibberellin	*gene45867*	4.98	*GA2ox*	Gibberellin 2-oxidase
	*gene22292*	2.79	*GA2ox*	Gibberellin 2-oxidase
	*gene12319*	2.68	*GA2ox*	Gibberellin 2-oxidase
	*gene14834*	2.47	*GA2ox*	Gibberellin 2-oxidase
	*gene56942*	1.91	*GA2ox*	Gibberellin 2-oxidase
	*gene55701*	1.81	*GA2ox*	Gibberellin 2-oxidase
	*gene53350*	1.60	*GA2ox*	Gibberellin 2-oxidase
	*gene11302*	1.57	*GA2ox*	Gibberellin 2-oxidase
	*gene40263*	–1.69	*GA2ox*	Gibberellin 2-oxidase
	*gene1876*	–1.99	*GA2ox*	Gibberellin 2-oxidase

There are many enzymes involved in the biosynthesis of gibberellin in plants. Interaction with strain YC0136 increased the expression level of *GA2ox* genes coding gibberellin-2-oxidase (GA2ox) in tobacco by 1.57–4.98 times (Table 1).

*Cis*-zeatin *O*-glucosyltransferase converts *cis*-zeatin to zeatin glycoside. Interaction with strain YC0136 caused the expression of several genes encoding *cis*-zeatin *O*-glucosyltransferase in tobacco to change by varying degrees (Supplementary Table S2). Seven genes (*gene51315*, *gene50*, *gene53518*, *gene68033*, *gene41639*, *gene48349*, and *gene24068*) were down-regulated following culture with strain YC0136. Changes in the expression levels of genes encoding *cis*-zeatin *O*-glucosyltransferase showed that accumulation of zeatin is an effective response to interactions with strain YC0136.

#### Strain YC0136 Enhances Systemic Resistance in Tobacco

After interacting with strain YC0136, *fls2* genes (*gene32927*, *gene40590*, and *gene40591*) of tobacco, coding flagellin-sensing 2 protein, were up-regulated by 1.87–3.35 times compared with those in tobacco roots not cultured with strain YC0136. This indicated that strain YC0136 may stimulate the early immune response in tobacco.

##### Strain YC0136 induces expression of transcription factors involved in plant stress tolerance

The WRKY transcriptional regulators belong to one of the largest transcriptional regulator families in plants. Interaction with strain YC0136 produced up-regulation of expression of some WRKY transcriptional regulator genes in tobacco (Table 2). Two genes (*gene2945* and *gene35472*) encoding WRKY2 were up-regulated by 3.35 and 2.09 times, respectively, compared with those in tobacco roots not cultured with strain YC0136. Genes encoding WRKY33 (*gene72767*, *gene6517*, *gene34934*, and *gene31508*) were up-regulated by 1.67, 1.90, 1.84, and 1.52 times, respectively.

**TABLE 2:** Effects of Strain YC0136 on the expression of transcription factors in tobacco.

Type	Gene ID	Log_2_(FC)	Gene	Product
WRKY	*gene2945*	3.35	*WRKY2*	WRKY transcription factor 2
	*gene35472*	2.09	*WRKY2*	WRKY transcription factor 2
	*gene6517*	1.90	*WRKY33*	WRKY transcription factor 33
	*gene34934*	1.84	*WRKY33*	WRKY transcription factor 33
	*gene72767*	1.67	*WRKY33*	WRKY transcription factor 33
	*gene31508*	1.52	*WRKY33*	WRKY transcription factor 33
HSF	*gene27131*	3.47	*HSF*	Heat shock transcription factor
	*gene62360*	2.47	*HSF*	Heat shock transcription factor
	*gene38294*	2.46	*HSF*	Heat shock transcription factor
	*gene23752*	2.46	*HSF*	Heat shock transcription factor
	*gene33933*	2.05	*HSF*	Heat shock transcription factor
	*gene38489*	1.78	*HSF*	Heat shock transcription factor
	*gene3507*	1.65	*HSF*	Heat shock transcription factor
	*gene9029*	−1.58	*HSF*	Heat shock transcription factor
MYB	*gene16596*	5.44	*MYB*	Transcription factor MYB
	*gene62785*	4.05	*MYB*	Transcription factor MYB
	*gene7813*	3.68	*MYB*	Transcription factor MYB
	*gene19987*	3.66	*MYB*	Transcription factor MYB
	*gene37246*	3.37	*MYB*	Transcription factor MYB
	*gene41931*	3.13	*MYB*	Transcription factor MYB
	*gene26405*	3.03	*MYB*	Transcription factor MYB
	*gene41368*	2.90	*MYB*	Transcription factor MYB
	*gene6921*	2.73	*MYB*	Transcription factor MYB
	*gene37631*	2.69	*MYB*	Transcription factor MYB
	*gene64315*	2.65	*MYB*	Transcription factor MYB
	*gene32699*	2.61	*MYB*	Transcription factor MYB
	*gene48183*	2.49	*MYB*	Transcription factor MYB
	*gene41932*	2.46	*MYB*	Transcription factor MYB
	*gene39829*	2.40	*MYB*	Transcription factor MYB
	*gene70785*	2.30	*MYB*	Transcription factor MYB
	*gene71054*	2.25	*MYB*	Transcription factor MYB
	*gene7811*	2.23	*MYB*	Transcription factor MYB
	*gene6521*	2.22	*MYB*	Transcription factor MYB
	*gene39956*	2.04	*MYB*	Transcription factor MYB
	*gene742*	1.92	*MYB*	Transcription factor MYB
	*gene26446*	1.90	*MYB*	Transcription factor MYB
	*gene18880*	1.88	*MYB*	Transcription factor MYB
	*gene60170*	1.55	*MYB*	Transcription factor MYB
	*gene15730*	1.52	*MYB*	Transcription factor MYB
MYC2	*gene5340*	1.73	*MYC2*	Transcription factor MYC2

Strain YC0136 caused changes in the expression of other transcription factors related to abiotic stress responses in plants, including heat shock transcription factors and MYB transcription factors. After interaction with strain YC0136, *gene27131*, *gene62360*, *gene38294*, *gene23752*, *gene33933*, *gene38489*, and *gene3507* encoding HSFs in tobacco were up-regulated by 1.6–3.47 times compared with those in tobacco roots not cultured with strain YC0136. Strain YC0136 induced the expression of genes encoding MYB transcription regulators in tobacco. These genes were up-regulated by 1.52–5.44 times compared with those in tobacco roots not cultured with strain YC0136.

The MYC2 transcriptional regulatory factor is the core of the plant jasmonic acid signaling pathway. After interaction with strain YC0136, *gene5340* encoding MYC2 in tobacco was up-regulated by 1.73 times.

##### Strain YC0136 induces expression of other genes related to systemic resistance in tobacco

Strain YC0136 can not only induce the expression of transcription factors related to stress tolerance, but also induce the systemic resistance of tobacco through the salicylic acid pathway and some secondary metabolites.

Salicylic acid is a widely recognized plant endogenous signaling molecule associated with a variety of plant resistances (Durner et al., 1997). In tobacco, we found that genes involved in the salicylic acid pathway were significantly up-regulated by strain YC0136 (Table 3). Expression of genes encoding SGT1 (pathogen-inducible salicylic acid glucosyltransferase) was significantly up-regulated compared with that in tobacco roots not cultured with strain YC0136. These nine genes (*gene10335*, *gene32550*, *gene32965*, *gene34783*, *gene42409*, *gene42410*, *gene64161*, *gene64162*, and *gene64163*) were up-regulated by 1.67–3.92 times.

**TABLE 3:** Effects of strain YC136 on salicylic acid signaling pathway in tobacco.

Gene ID	Log_2_ (FC)	Gene	Product
*gene32550*	3.92	*SGT1*	Pathogen-inducible salicylic acid glucosyltransferase
*gene64162*	3.91	*SGT1*	Pathogen-inducible salicylic acid glucosyltransferase
*gene64163*	3.85	*SGT1*	Pathogen-inducible salicylic acid glucosyltransferase
*gene64161*	3.5	*SGT1*	Pathogen-inducible salicylic acid glucosyltransferase
*gene42409*	3.14	*SGT1*	Pathogen-inducible salicylic acid glucosyltransferase
*gene10335*	3.05	*SGT1*	Pathogen-inducible salicylic acid glucosyltransferase
*gene32965*	2.18	*SGT1*	Pathogen-inducible salicylic acid glucosyltransferase
*gene34783*	1.71	*SGT1*	Pathogen-inducible salicylic acid glucosyltransferase
*gene42410*	1.67	*SGT1*	Pathogen-inducible salicylic acid glucosyltransferase

Many secondary metabolites can enhance the resistance of plants to diseases. After inoculating with strain YC0136, some genes involved in the phenylpropanoid metabolic pathway in tobacco were up-regulated by varying degrees compared with those in tobacco roots not cultured with strain YC0136 (Table 4). Phenylalanine ammonia-lyase (PAL) is a key enzyme in the metabolic pathway of phenylpropanoids. The genes encoding PAL, *gene23596*, *gene50105*, *gene55404*, *gene63885*, *gene13924*, and *gene2736*, were up-regulated by 1.51–3.26 times following culture with strain YC0136. Four genes encoding cinnamic acid 4-hydroxylase (C4H) were up-regulated by 1.68–4.39 times, and the genes encoding shikimic acid *O*-hydroxycinnamoyl transferase, *gene52491*, *gene59014*, and *gene68771*, were up-regulated by 1.50–1.96 times. The expression of genes encoding caffeic acid 3-*O*-methyltransferase (COMT) (*gene49647*, *gene51252*, and *gene57715*) was up-regulated by 2.26–4.59 times, while nine genes related to caffeic acid CoA-*O*-methyl transferase (CCoAOMT) were up-regulated by 1.63–3.27 times. The up-regulation of genes encoding 4-coumarin-CoA ligase following culture with strain YC0136 was not significant.

**TABLE 4:** Effects of Strain YC0136 on genes related to phenylpropane compound metabolism.

Gene ID	Log_2_ (FC)	Gene	Product
*gene48890*	1.75	*4CL*	4-Coumaroyl-CoA ligase
*gene31505*	1.66	*4CL*	4-Coumaroyl-CoA ligase
*gene65338*	1.65	*4CL*	4-Coumaroyl-CoA ligase
*gene59318*	1.63	*4CL*	4-Coumaroyl-CoA ligase
*gene14196*	4.39	*C4H*	Cinnamic acid 4-hydroxylase
*gene45704*	3.85	*C4H*	Cinnamic acid 4-hydroxylase
*gene16642*	2.66	*C4H*	Cinnamic acid 4-hydroxylase
*gene73634*	1.68	*C4H*	Cinnamic acid 4-hydroxylase
*gene49647*	4.59	*COMT*	Caffeic acid 3-*O*-methyltransferase
*gene51252*	2.27	*COMT*	Caffeic acid 3-*O*-methyltransferase
*gene57715*	2.26	*COMT*	Caffeic acid 3-*O*-methyltransferase
*gene52406*	2.61	*CCoAOMT*	Caffeic acid CoA-*O*-methyl transferase
*gene52407*	3.27	*CCoAOMT*	Caffeic acid CoA-*O*-methyl transferase
*gene48930*	2.95	*CCoAOMT*	Caffeic acid CoA-*O*-methyl transferase
*gene48931*	2.74	*CCoAOMT*	Caffeic acid CoA-*O*-methyl transferase
*gene43754*	2.2	*CCoAOMT*	Caffeic acid CoA-*O*-methyl transferase
*gene9422*	1.86	*CCoAOMT*	Caffeic acid CoA-*O*-methyl transferase
*gene59406*	1.74	*CCoAOMT*	Caffeic acid CoA-*O*-methyl transferase
*gene71741*	1.73	*CCoAOMT*	Caffeic acid CoA-*O*-methyl transferase
*gene37383*	1.63	*CCoAOMT*	Caffeic acid CoA-*O*-methyl transferase
*gene52491*	1.96	*HCT*	Shikimic acid *O*-hydroxycinnamoyl transferase
*gene59014*	1.88	*HCT*	Shikimic acid *O*-hydroxycinnamoyl transferase
*gene68771*	1.50	*HCT*	Shikimic acid *O*-hydroxycinnamoyl transferase
*gene48554*	–2.57	*HCT*	Shikimic acid *O*-hydroxycinnamoyl transferase
*gene213*	–3.53	*HCT*	Shikimic acid *O*-hydroxycinnamoyl transferase
*gene23596*	3.26	*PAL*	Phenylalanine ammonia-lyase
*gene50105*	2.96	*PAL*	Phenylalanine ammonia-lyase
*gene55404*	2.59	*PAL*	Phenylalanine ammonia-lyase
*gene63885*	2.53	*PAL*	Phenylalanine ammonia-lyase
*gene13924*	2.01	*PAL*	Phenylalanine ammonia-lyase
*gene2736*	1.51	*PAL*	Phenylalanine ammonia-lyase

Laccase plays an important role in plant resistance to insects and fungi. The expression levels of genes encoding laccase were up-regulated by 1.85–4.81 times in tobacco roots cultured with strain YC0136 compared with those in tobacco roots not cultured with strain YC0136 (Supplementary Table S3). Up-regulation of laccase genes is helpful for enhancing the toughness of the tobacco rhizome, increasing the resistance of tobacco to pests and pathogens.

##### Strain YC0136 affects the expression of genes involved in tobacco metabolic pathways

After interacting with strain YC0136, some genes related to metabolism in tobacco were expressed differently. There were 51 DEGs related to amino acid metabolism (Supplementary Table S4). A total of 37 genes were up-regulated, while 14 genes were down-regulated following culture with strain YC0136. *gene51112* encoding branched-chain amino acid transaminase was up-regulated by 5.76 times compared with that in tobacco roots not cultured with strain YC0136. In addition, *gene57308* and *gene9555*, encoding asparagine synthetase involved in glutamine hydrolysis, were up-regulated by 5.0 and 3.96 times, respectively. Among the 14 down-regulated genes, *gene42183* encoding glutamine synthetase was down-regulated by 1.96 times following culture with strain YC0136. *gene60280* encoding threonine synthetase was down-regulated by 2.21 times. These results indicated that strain YC0136 can reduce glutamine content in tobacco by inducing glutamine degradation and inhibiting its biosynthesis.

There were 183 DEGs related to glycol metabolism, of which 71 genes were up-regulated and 112 genes were down-regulated following culture with strain YC0136 (Supplementary Table S5). Among the up-regulated genes, *gene66000* and *gene1858*, which encode the reciprocal transformation of pentose and glucuronic acid, were up-regulated by 6.80 and 5.26 times, respectively, compared with those in tobacco roots not cultured with strain YC0136. Two genes, *gene27593* and *gene47222*, involved in the metabolism of cyanidin-3-glucoside, were up-regulated by 5.14 and 4.54 times, respectively. Strain YC0136 also induced the expression of the gene (*gene458809*) encoding xylan 1,4-β-xylosidase, which was up-regulated by 1.56 times compared with that in tobacco roots not cultured with strain YC0136. In plants, β-fructofuranosidase is involved in the conversion of sucrose. The expression of genes (*gene69223*, *gene6072*, and *gene53789*) encoding β-fructofuranosidase was down-regulated by 4.44–4.54 times following culture with strain YC0136. These results indicated that strain YC0136 attenuates the sucrose conversion ability of tobacco.

#### Effects of Tobacco on Gene Expression in Strain YC0136

##### Tobacco stimulates the expression of auxin biosynthesis genes and motility genes in strain YC0136

Indole-3-pyruvate decarboxylase (IPDC) is a key enzyme in the *IPyA* pathway of auxin biosynthesis, which converts indole-3-pyruvic acid into indole-3-acetaldehyde (Malhotra and Srivastava, 2008). The similarity in protein sequence between *ilvB* (*PPYC1_16985*) of strain YC0136 and *ipdC* of *P. polymyxa* E681 was 100%. Gene *PPYC1_23850* encodes aldehyde dehydrogenase, which converts indole-3-acetaldehyde into IAA. Under conditions of interaction with tobacco roots, expression of the *ilvB* gene in strain YC0136 was up-regulated by 1.72 times compared with that under conditions without tobacco roots, and expression of gene *PPYC1_23850* was up-regulated by 1.12 times. This indicated that tobacco may promote auxin biosynthesis in strain YC0136. The flagellum motor switch is related to bacterial motility. Under interaction conditions, the expression of the flagellum motor switch gene *fliG* (*PPYC1_14375*) in strain YC0136 was up-regulated by 1.41 times compared with that in strain YC0136 without tobacco interaction. The transcription of genes encoding flagellum assembly proteins, *fliH* (*PPYC1_14370*) and *fliF* (*PPYC1_14380*), was up-regulated by 1.34 times and 1.54 times, respectively, following interaction with tobacco.

##### Tobacco affects the transporter genes of strain YC0136

After interacting with tobacco, genes related to transport in strain YC0136 were changed by varying degrees (Table 5). There were 29 genes up-regulated compared with their expression levels without tobacco interaction. Among them, 16 genes related to ABC transport were up-regulated by 1.59–4.32 times. The operon *pstSABC*, which is involved in inorganic phosphorus transport, was up-regulated 3.78–4.32 times. Three genes (*PPYC1_06875*, *PPYC1_15095*, and *PPYC1_19620*) belonging to the MFS transport family were up-regulated by 1.57, 4.34, and 2.20 times, respectively. The expression levels of partial genes in strain YC0136 were down-regulated following interaction with tobacco roots, compared with those in strain YC0136 without tobacco interaction. Four genes involved in ABC transport were down-regulated by 1.54–2.60 times. Two genes (*PPYC1_19055* and *PPYC1_22815*) involved in MFS transfer were down-regulated by 1.67 times and 4.69 times, respectively. These benefit the growth of strain YC0136 by absorbing nutrients.

**TABLE 5:** Effects of tobacco on the genes related to transport in strain YC0136.

Gene ID	Log_2_ (FC)	Name	Product
*PPYC1_17625*	2.89		Metal ABC transporter permease
*PPYC1_17630*	2.78		Manganese ABC transporter
*PPYC1_00910*	1.6		Nickel ABC transporter permease subunit NikC
*PPYC1_17640*	1.94		Manganese transporter
*PPYC1_03855*	2.11	*phnD*	Phosphate/phosphite/phosphonate ABC transporter substrate-binding protein
*PPYC1_15975*	3.78	*pstB*	Phosphate ABC transporter ATP-binding protein
*PPYC1_03845*	1.75	*phnE*	Phosphonate ABC transporter, permease protein PhnE
*PPYC1_00905*	1.72		Nickel ABC transporter permease subunit NikB
*PPYC1_17635*	2.56		Metal ABC transporter ATP-binding protein
*PPYC1_15980*	3.96	*pstA*	Phosphate ABC transporter, permease protein PstA
*PPYC1_15985*	3.93	*pstC*	Phosphate ABC transporter permease subunit PstC
*PPYC1_15910*	4.32	*pstB*	Phosphate ABC transporter ATP-binding protein
*PPYC1_15990*	4.16	*pstS*	Phosphate ABC transporter substrate-binding protein PstS
*PPYC1_06875*	1.57	*oxlT*	MFS transporter
*PPYC1_15095*	4.34		MFS transporter
*PPYC1_19620*	2.2		MFS transporter
*PPYC1_16575*	1.67		Lantibiotic ABC transporter permease
*PPYC1_20185*	2.22		Glutamine ABC transporter permease
*PPYC1_06535*	1.5		Efflux RND transporter periplasmic adaptor subunit
*PPYC1_03665*	1.75	*arsB*	Arsenic transporter
*PPYC1_15275*	3.31		Ammonium transporter
*PPYC1_18230*	1.91		ABC transporter permease
*PPYC1_16580*	1.59		ABC transporter permease
*PPYC1_02025*	–1.54		Amino acid ABC transporter substrate-binding protein
*PPYC1_01710*	–1.58		ABC transporter permease
*PPYC1_19055*	–1.67	*araE*	MFS transporter
*PPYC1_00995*	–1.72		PTS sugar transporter subunit IIB
*PPYC1_13110*	–2.04	*znuB*	Metal ABC transporter permease
*PPYC1_13115*	–2.6	*znuC*	Metal ABC transporter ATP-binding protein
*PPYC1_00515*	–3.13	*smr*	QacE family quaternary ammonium compound efflux SMR transporter
*PPYC1_22815*	–4.69		MFS transporter

To summarize, *P. polymyxa* YC0136 isolated from the rhizosphere of tobacco directly promoted the growth of tobacco by secreting auxin and solubilizing phosphate. Strain YC0136 also promoted the growth of tobacco by inducing the expression of genes related to plant hormone signal transduction in tobacco. Strain YC0136 antagonized pathogens by secreting secondary metabolites, and also induced systemic resistance in tobacco. This study contributes to our understanding of the growth-promoting mechanisms of strain YC0136 on tobacco and provides a theoretical basis for the application of *P. polymyxa* YC0136 as a biological fertilizer.

## Discussion

This study was carried out to understand the promotion mechanism of strain YC0136 on improving the agronomic traits of tobacco and enhancing the resistance of tobacco. We found strain YC0136 can antagonize pathogenic bacteria and fungi. In our previous report (Liu et al., 2017), we used antiSMASH database to analyze the whole genome of strain YC0136 and found there were 14 gene clusters related to secondary metabolites controlling pathogenic bacteria and fungi. The existence of these gene clusters may cause the ability of strain YC0136 controlling pathogens. The results of pot experiments indicated that strain YC0136 can improve the agronomic characteristics of tobacco.

The transcriptome of tobacco roots during interaction with strain YC0136 revealed that strain YC0136 can stimulate auxin, gibberellin and cytokinin signal transduction in tobacco. Gibberellin-2-oxidase (GA2ox) is a key enzyme that plays a negative regulatory role in the gibberellin biosynthesis pathway (Wuddineh et al., 2015). Interaction with strain YC0136 increased the expression level of *GA2ox* genes in tobacco. Up-regulated expression of *GA2ox* can reduce gibberellin content and improve plant architecture (Wuddineh et al., 2015). High expression levels of genes encoding GA2ox could increase the response of tobacco to abiotic stress (Colebrook et al., 2014). In addition, gibberellin catabolism helps plants respond to adverse conditions, such as drought and high salinity (Zawaski and Busov, 2014; Zhou and Underhill, 2016). These results indicate that strain YC0136 might be beneficial to plant growth and promote plant stress tolerance. Zeatin is a natural cytokinin in plants that promotes cell division and delays plant senescence (Martin et al., 1999, 2001). Strain YC0136 attenuated the ability of zeatin conversion in tobacco by inhibiting the expression of *cis*-zeatin *O*-glucosyltransferase. This reduced the degradation of zeatin, allowing zeatin to play a role as a cytokinin (Kudo et al., 2012; Shang et al., 2016).

After interacting with strain YC0136, a large number of genes in tobacco were expressed differently. Flagellin-sensing 2 (FLS2) plays an important role in plant and microbial recognition (Chinchilla et al., 2007). FLS2 interacts with 22 N-terminal conserved amino acids in bacterial flagellin to induce plant defense responses. In *Arabidopsis thaliana fls2* mutant, expression of *VvFLS2* could restore flg22-induced H_2_O_2_ production, and *VvFLS2* could induce grapevine immune response to *Botrytis cinereal* (Trdá et al., 2014). In tobacco interacted with strain YC0136, *fls2* was up-regulated. We speculated that FLS2 protein stimulated the immune response of tobacco through recognizing with flg22 peptides in strain YC0136. Strain YC0136 may also overcome MAMP-triggered immunity to colonize plants as *Burkholderia phytofirmans* (Trdá et al., 2014). Transcriptional regulatory factors have extremely important effects on the growth and development of organisms (Yamaguchi-Shinozaki and Shinozaki, 2006; Pankaj et al., 2015; Lin et al., 2017). WRKY proteins are plant-specific transcriptional regulatory factors involved in plant growth and seed development (Ay et al., 2009; Jiang and Yu, 2009), plant disease resistance (Dang et al., 2013; Wang et al., 2013), plant signal transduction (Yang et al., 1999; Antoni et al., 2011; Peng et al., 2012), and abiotic stress (Zou et al., 2010; Sun et al., 2015). Mzid et al. (2007) reported that overexpression of *VvWRKY2* in tobacco could enhance broad resistance to necrotrophic fungal pathogens. In this study, we found that *gene2945* in tobacco, encoding transcription regulator WRKY2, was up-regulated by 3.35 times following culture with strain YC0136, compared with its expression level without strain YC0136. This is conducive to enhancing tobacco resistance to pathogens. Another transcription regulator in plants, WRKY33, also plays an important role in plant growth, development, and other life activities. WRKY33 can not only enhance the tolerance of plants to salt stress (Jiang and Deyholos, 2009; Jiang et al., 2014), but also plays an essential role in defense against necrotrophic fungi (Birkenbihl et al., 2012). After interacting with strain YC0136, the genes encoding WRKY33 were also up-regulated in tobacco. This indicates that strain YC0136 may induce tobacco plants to develop systemic resistance to better respond to abiotic and biological stresses.

In the transcriptome of tobacco interacted with stain YC0136, the genes of PAL, 4CL, COMT, CCoAOMT, shikimic acid *O*-hydroxycinnamoyl transferase, and C4H were up-regulated in varying degrees. The six enzymes mentioned above are all involved in the biosynthesis of phenylpropanoids (Ferrer et al., 2008). Phenylpropanoids are secondary metabolites biosynthesized from phenylalanine in plants and they can enhance the ability of plants to resist various biotic and abiotic stresses (Ye and Varner, 1995; Toquin et al., 2003; Korkina, 2007; Yu and Jez, 2008; Liu et al., 2016; Zhang et al., 2020). Chen et al. reported that overexpression of *LjPAL* could enhance the resistance to *Pseudomonas syringae* pv. *phaseolicola* NPS3121 infection in *L. japonicus* (Chen et al., 2017). Yamamoto et al. (1998) reported that the activity of PAL was increased in tobacco treated with phosphorus starvation and it protected tobacco cells from the cytotoxic lipid peroxidation caused by the combination of aluminum and iron. In wheat, overexpression of TaCOMT-3D can enhance the resistance of wheat to sharp eyespot disease (Wang et al., 2018). The overexpression of *4CL* and *STS* in tobacco could not only enhance the antagonistic activity against the pathogen *Monilinia fructicola*, but also significantly enhance the tolerance to salt stress (He et al., 2018). In previous studies, pathogens can induce the up-regulation of PAL and other related enzymes genes in plants. In this study, although strain YC0136 was a beneficial bacteria, the PAL, COMT and other enzyme genes of tobacco interacted with strain YC0136 were also up-regulated. The results indicated that strain YC0136 could induce the systemic resistance of tobacco by stimulating phenylpropanoids metabolism in the absence of pathogenic bacteria and adversity. Besides, enzymes of PAL, COMT and CCoAOMT are also involved in different methylation pathways in lignin biosynthesis of plants (Ye and Varner, 1995; Chen et al., 2017). The up-regulation of PAL, COMT, and CCoAOMT might increase the content of lignin in tobacco. Lignin is an important part of secondary thickening of cell wall, it can enhance mechanical strength of plants, and protect plant against insects and pathogens (Dauwe et al., 2010). The accumulation of lignin helps to enhance the mechanical strength of tobacco, thus enhancing the resistance of tobacco to pests and pathogens.

Tobacco also affected the growth of strain YC0136 under conditions of their interaction. In strain YC0136, the expression of genes (*ilvB* and *PPYC1_23850*) involved in auxin biosynthesis was up-regulated in the presence of tobacco roots. This indicates that materials in root exudates may induce auxin biosynthesis in strain YC0136. Kwon et al. (2016) also reported that the interaction between *P. polymyxa* E681 and *Arabidopsis thaliana* results in the enhancement of auxin secretion in *P. polymyxa* E681. The expression of genes related to the phosphate-specific transport system (*pstSCAB*, *phoU*) was significantly up-regulated by interaction with tobacco roots. The *pst* operon is a high-affinity phosphate transport system (Qi et al., 1997; Aguena et al., 2002). The up-regulation of *pst* operon expression is beneficial to meet the phosphorus requirement and promote the growth of strain YC0136. The transcriptome revealed that 31 genes related to transport were expressed differently in strain YC0136 in the presence of tobacco roots. These genes are related to the transport of metal ions, phosphates, glutamine, arsenic, and ammonium. They are beneficial for enhancing the absorption of inorganic salt ions and the growth of strain YC0136.

**FIGURE 7:**
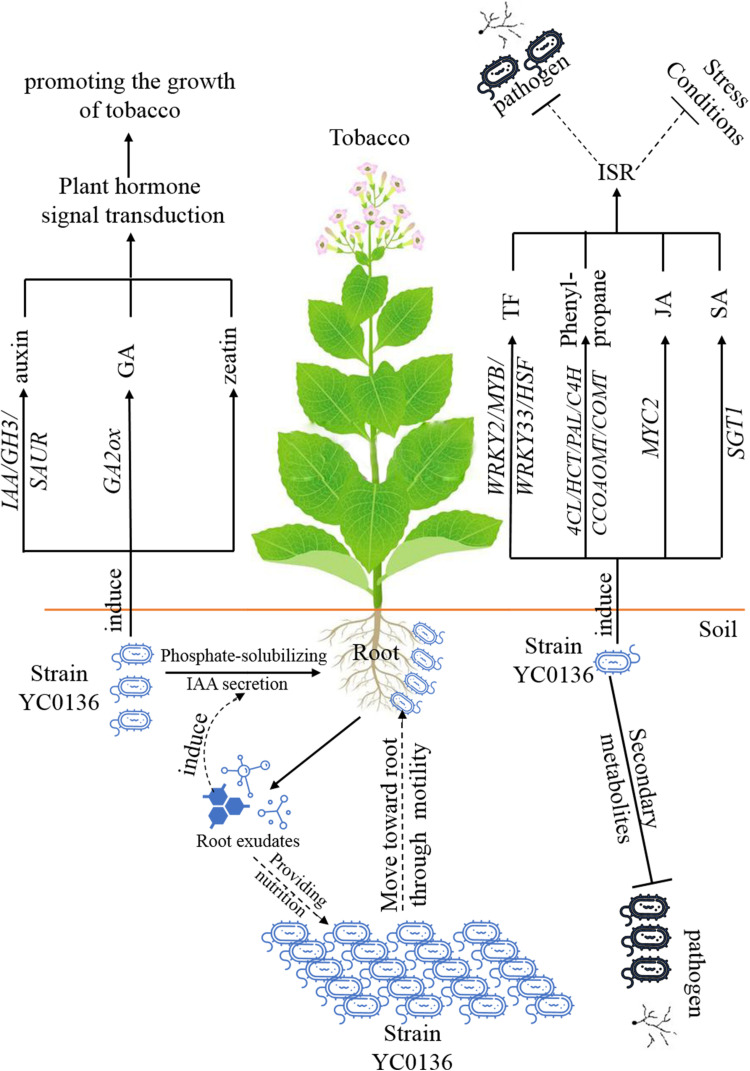
Proposed model of the growth-promoting mechanisms of strain YC0136 on tobacco.

We summarize the relationship between strain YC0136 and tobacco in a pattern diagram, as shown in Figure 7. Tobacco root exudates may attract strain YC0136, then YC0136 cells move toward the tobacco root through flagella rotation. The tobacco root exudates may provide nutrients for the growth of strain YC0136. Strain YC0136 might promote the growth of tobacco by secreting auxin and solubilizing phosphate. At the same time, strain YC0136 promotes the growth of tobacco by inducing auxin, gibberellin, and zeatin signaling. Strain YC0136 biosynthesizes secondary metabolites to antagonize pathogens. Strain YC0136 also induces systemic resistance of tobacco by stimulating the expression of transcriptional regulatory factors (such as WRKY and MYB, etc.) and related genes of the phenylpropanoid biosynthesis pathway.

## Data Availability Statement

The datasets presented in this study can be found in online repositories. The names of the repository/repositories and accession number(s) can be found in the article/Supplementary Material.

## Author Contributions

CW, YD, and BD designed the study. HL, JW, and HS performed the laboratory work and analyzed the data. HL wrote the manuscript. KL, XH, YP, and JL advised the manuscript. YD, BD, and CW supported the study. All authors contributed to the article and approved the submitted version.

## Conflict of Interest

The authors declare that the research was conducted in the absence of any commercial or financial relationships that could be construed as a potential conflict of interest.

## Footnotes

1 www.ncbi.nlm.nih.gov/nuccore/AYMY00000000.1/

2 http://www.genome.jp/kegg
